# Evidence-based production framework for herbal medicine regulation in Indonesia

**DOI:** 10.3389/fphar.2025.1730273

**Published:** 2026-01-02

**Authors:** Mohamad Kashuri, Taruna Ikrar, Sutriyo, Abdul Mun’im, Arry Yanuar

**Affiliations:** 1 Faculty of Pharmacy Universitas Indonesia, Depok, West Java, Indonesia; 2 The Indonesian Food and Drug Authority, Jakarta, Indonesia

**Keywords:** ASEAN harmonization, evidence-based production, herbal medicine regulation, omics authentication, pharmacovigilance, quality by design, regulatory science

## Abstract

This narrative review synthesizes 2015–2025 evidence on evidence-based production (EBP) of herbal medicines with emphasis on advanced production technologies, omics-enabled authentication, quality by design (QbD), and regulatory harmonization relevant to Indonesia. We map how *in vitro* root culture, bioreactor scale-up, elicitation/metabolic engineering, and nanotechnology address supply variability and improve consistency; how DNA barcoding/metabarcoding and metabolomics with chemometrics underpin identity and chemical reproducibility; and how ASEAN/WHO initiatives enable ‘loose harmonization’ while preserving traditional diversity. We argue for a two-key batch-release specification (genomic × metabolite) and validated omics workflows within GLP to strengthen traceability, with real-world evidence and digital pharmacovigilance extending safety monitoring post-market. We translate these elements into an actionable framework for the Indonesian FDA (BPOM) to operationalize EBP through regulation, cross-sector training, and reliance pathways, positioning Indonesia as a regional hub for evidence-based herbal regulation.

## Graphical Abstract

**Figure:**
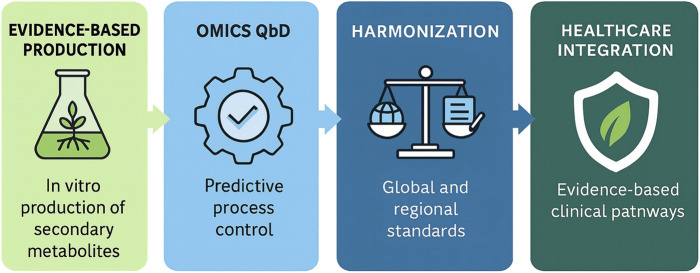

## 1 Introduction

Herbal medicines have regained prominence worldwide as accessible, culturally rooted therapeutic options, driven by sustained consumer demand and interest in complementary care (Knoess and Wiesner, 2019; Raclariu-Manolică et al., 2023). This resurgence, however, has exposed persistent weaknesses in quality assurance: product heterogeneity, species misidentification, adulteration, and inconsistent evidence of efficacy all undermine patient safety and regulatory confidence (De Boer et al., 2015; Soares and Ferreira, 2017). At the same time, rapid biotechnological and analytical advances ranging from *in vitro* adventitious-root production and scalable bioreactors to nanocarrier formulations and high-throughput molecular authentication offer concrete pathways to overcome supply instability and compositional variability (Awlqadr et al., 2025; Da Silva et al., 2021; Hosseini et al., 2024; Srinath et al., 2022).

Despite these scientific gains, regulatory frameworks remain fragmented across jurisdictions, impeding mutual recognition, trade, and robust post-market surveillance (Knoess and Wiesner, 2019; Qu et al., 2018). Pharmacopoeial differences, variable implementation of good agricultural and collection practices (GACP)–good manufacturing practices (GMP)–good laboratory practices (GLP) principles, and limited uptake of omics-enabled quality metrics create a disconnect between laboratory evidence and policy action (Kauffmann et al., 2017). Moreover, much of the literature to date treats production technologies, molecular authentication, and regulatory policy as discrete domains rather than components of a coherent evidence-to-policy pipeline that regulators can operationalize (Raclariu-Manolică et al., 2023; Dubale et al., 2025).

This review addresses that translational gap by synthesizing recent advances in production technologies (e.g., *in vitro* root culture, bioreactor scale-up, metabolic engineering, and nanotechnology), omics-based authentication deoxyribonucleic acid (DNA) barcoding/metabarcoding and metabolomics with chemometrics), and contemporary approaches to regulatory harmonization and pharmacovigilance (De Boer et al., 2015; Dev et al., 2021; García-Pérez et al., 2024). Building on world health organization (WHO) and regional initiatives, the paper frames how evidence generated in laboratories can be translated into practical regulatory criteria such as a dual “DNA × metabolite” batch-release standard and validated omics workflows that strengthen product traceability and safety monitoring (Broojerdi et al., 2024; Hölzle et al., 2024; Raclariu et al., 2018).

The specific objectives are to (1) map contemporary scientific tools that improve reproducibility and authenticity of herbal products; (2) evaluate policy mechanisms and harmonization models relevant to Indonesia and ASEAN; and (3) propose an actionable, evidence-based regulatory framework tailored to the Indonesian Food and Drug Authority/Badan Pengawas Obat dan Makanan (BPOM) mandate. The review asks three focused questions: How can omics and advanced production systems be integrated into regulatory quality criteria? What harmonization strategies enable practical alignment across pharmacopeias while respecting traditional diversity? And which institutional steps will operationalize evidence-to-policy translation in national regulatory practice?

Novelty of this work lies in its integrative regulatory-science perspective: rather than separately cataloging technological advances or policy options, it synthesizes molecular authentication, omics-driven quality-by-design, and harmonization strategies into a unified framework aimed at operationalizing evidence-based production within national regulation. By doing so, the review furnishes the BPOM and regional partners with a practicable roadmap to institutionalize scientific evidence in herbal-medicine oversight, enhancing product safety, market credibility, and public health protection (Raclariu-Manolică et al., 2023; Kauffmann et al., 2017; Dubale et al., 2025).

## 2 Methods

This study adopted a structured narrative review design to synthesize scientific and regulatory evidence on the evidence-based production (EBP) of herbal medicines within the Indonesian and ASEAN contexts. The approach emphasized conceptual integration, policy relevance, and methodological transparency rather than quantitative meta-analysis, consistent with narrative synthesis principles in regulatory science. Literature searches were performed across Scopus, PubMed, Web of Science, and WHO IRIS databases, covering publications from 2015 to 2025. Keyword combinations included “evidence-based production,” “herbal medicine regulation,” “DNA barcoding,” “metabolomics,” “quality by design,” and “pharmacovigilance,” refined using Boolean operators (AND, OR) to ensure search precision.

Inclusion criteria comprised peer-reviewed journal articles, official WHO or ASEAN regulatory documents, and pharmacopoeial guidelines published in English. Non-scientific commentaries, duplicate records, single-case reports, and papers lacking methodological rigor were excluded. Screening and selection were conducted by the author, based on title, abstract, and full-text relevance to production technologies, quality assurance, or regulatory frameworks. The final body of literature was organized into four thematic domains: (1) modernized, quality-assured production systems, (2) omics-based quality control and authentication, (3) regulatory harmonization and policy frameworks, and (4) integration into healthcare systems and pharmacovigilance.

A thematic synthesis approach was applied to identify conceptual linkages, regulatory gaps, and implications for Indonesia’s policy transformation. Although not a systematic review, this narrative synthesis follows a transparent and reproducible framework. A summary of databases, search terms, and thematic classification is presented in Table 1 to enhance methodological clarity. This review used only published sources and involved no human or animal subjects; ethics approval was not required.

**TABLE 1:** Summary of search databases, keywords, and thematic classification.

Thematic domain	Representative keywords	Example sources/Databases	Analytical focus
Advanced Production Technologies	“*in vitro* root culture,” “bioreactor herbal production,” “metabolic engineering,” “nanotechnology herbal medicine”	Scopus, PubMed	Biotechnological methods for sustainable, scalable herbal production
Omics-Based Quality Control and Authentication	“DNA barcoding,” “metabolomics,” “chemometrics,” “quality by design (QbD)”	Web of Science, Scopus	Molecular and chemical authentication for regulatory traceability
Regulatory Harmonization and Policy Frameworks	“herbal regulation,” “pharmacopoeia comparison,” “ASEAN harmonization,” “WHO IRCH”	WHO IRIS, Scopus	Global–regional policy convergence and institutional capacity-building
Integration into Healthcare Systems and Pharmacovigilance	“pharmacovigilance,” “real-world evidence (RWE),” “digital health monitoring,” “safety of herbal medicines”	PubMed, WHO IRIS	Clinical integration, safety surveillance, and public trust mechanisms

This table summarizes the databases, search terms, and thematic domains used in the structured narrative review covering 2015–2025. It outlines how the literature was grouped into production technologies, omics-based quality control, regulatory frameworks, and healthcare integration.

Abbreviations: QbD, quality by design; PV, pharmacovigilance.

## 3 Advanced production technologies

Advancements in evidence-based production (EBP) have contributed to improved quality in the herbal medicine sector, particularly through approaches such as *in vitro* propagation, small-scale controlled cultivation, post-harvest quality optimization, and targeted enhancement of active constituents using low-cost elicitation methods. These context-appropriate production enhancements represent a shift toward more consistent, sustainable, and quality-assured models that support public health protection while remaining aligned with the foundational principles of traditional medicine.

*In vitro* adventitious root culture has emerged as one of the most reliable and environmentally sustainable biotechnological tools for producing high-value secondary metabolites from medicinal plants. This method allows controlled root induction from non-root tissues, ensuring biochemical uniformity and eliminating seasonal or geographical variability in phytochemical yield (Hosseini et al., 2024; Goel et al., 2024). The controlled nutrient supply and aseptic environment enable stable production of bioactive compounds such as eurycomanone, phenolic acids, and alkaloids (Rahmat and Kang, 2019). For example, *Pfaffia glomerata* and *Valeriana jatamansi* cultures have successfully yielded pharmacologically active metabolites comparable in quality to field-grown plants, while simultaneously reducing dependence on wild harvesting (Da Silva et al., 2021; Devi et al., 2021). These findings confirm that adventitious-root culture can serve as a sustainable alternative for large-scale production, supporting biodiversity conservation and reliable supply chains.

Scaling up these cultures requires optimization through bioreactor technology, which ensures reproducibility and economic feasibility. Controlled systems such as air-lift, stirred-tank, and mist bioreactors have proven effective in regulating aeration, nutrient distribution, and shear stress, resulting in enhanced metabolite accumulation (Murthy et al., 2016). Integrating elicitation strategies by applying abiotic or biotic stressors such as methyl jasmonate or ethrel stimulates the biosynthetic pathways responsible for secondary metabolite formation (Martínez-Chávez et al., 2024). Moreover, metabolic engineering approaches, including pathway modification and transcriptomic regulation, further boost productivity and quality (Martínez-Chávez et al., 2024; Xu et al., 2024). The synergy of these techniques has enabled efficient production of bioactive compounds like ginsenosides, eurycomanone, and andrographolide (Xu et al., 2024; Wang et al., 2024). Nevertheless, operational challenges remain, particularly concerning reactor design optimization and cost-effectiveness in industrial-scale applications (Azmi et al., 2015). The comparative contributions of each production technology to the evidence-based framework are summarized in Table 2, highlighting their respective roles in enhancing sustainability, consistency, and therapeutic reliability.

**TABLE 2:** Comparative framework of advanced production technologies in herbal medicine.

Technology cluster	Scientific focus	Innovation mechanism	Regulatory/Production benefit	Strategic outcome (EBP pillar)
*In vitro* Adventitious Root Culture	Cellular-level propagation for metabolite synthesis	Controlled induction and differentiation of roots from explants under aseptic, nutrient-optimized conditions	Ensures genetic stability, reproducibility of active compounds, and minimizes environmental harvesting	Sustainability and Source Reliability
Bioreactor Scale-Up	Transition from lab-scale to industrial production	Integration of fed-batch systems, aeration control, and nutrient circulation	Achieves scalable production with consistent yield and reduced contamination risk	Consistency and Scalability
Elicitation and Metabolic Engineering	Enhancement of biosynthetic pathways	Application of biotic/abiotic stressors and genetic regulation of metabolite pathways	Increases specific secondary metabolite concentration and optimizes biosynthetic efficiency	Potency and Productivity
Nanotechnology Integration	Formulation and delivery optimization	Use of nanocarriers (liposomes, nanoemulsions, lipid carriers) to stabilize and target phytochemicals	Improves bioavailability, controlled release, and pharmacokinetic predictability	Therapeutic Efficacy and Safety
Bioinformatics and System Biology Linkage	Integration of data-driven control	Modelling metabolic flux and pathway optimization using omics data	Enables predictive process control and adaptive regulatory compliance	Evidence Transparency and Process Validation

This table compares major production technologies adventitious-root culture, bioreactor systems, elicitation, metabolic engineering, and nanotechnology highlighting their scientific focus, innovation mechanisms, and contributions to evidence-based production.

Abbreviations: EBP, Evidence-Based Production.

In parallel, nanotechnology applications have revolutionized herbal formulation and delivery systems, addressing long-standing issues of stability, solubility, and bioavailability. Nanocarriers such as liposomes, solid lipid nanoparticles, and nanoemulsions improve pharmacokinetic performance and protect labile phytochemicals from oxidative or enzymatic degradation (Awlqadr et al., 2025; Patadiya et al., 2025; Shama, 2024). These systems enable targeted delivery to specific tissues, reducing dosage requirements and enhancing therapeutic outcomes (Amjad and Mahdi, 2023; Shree et al., 2025). For example, nanostructured formulations of antidiabetic and cardioprotective plant extracts have shown significantly higher clinical efficacy compared to conventional preparations (Bhadouria et al., 2025; Noor et al., 2025). Despite these advances, challenges persist regarding scalability, long-term toxicity, and regulatory standardization for nanophytomedicine approval (Majee et al., 2025; Rahman et al., 2025).

**FIGURE 1:**
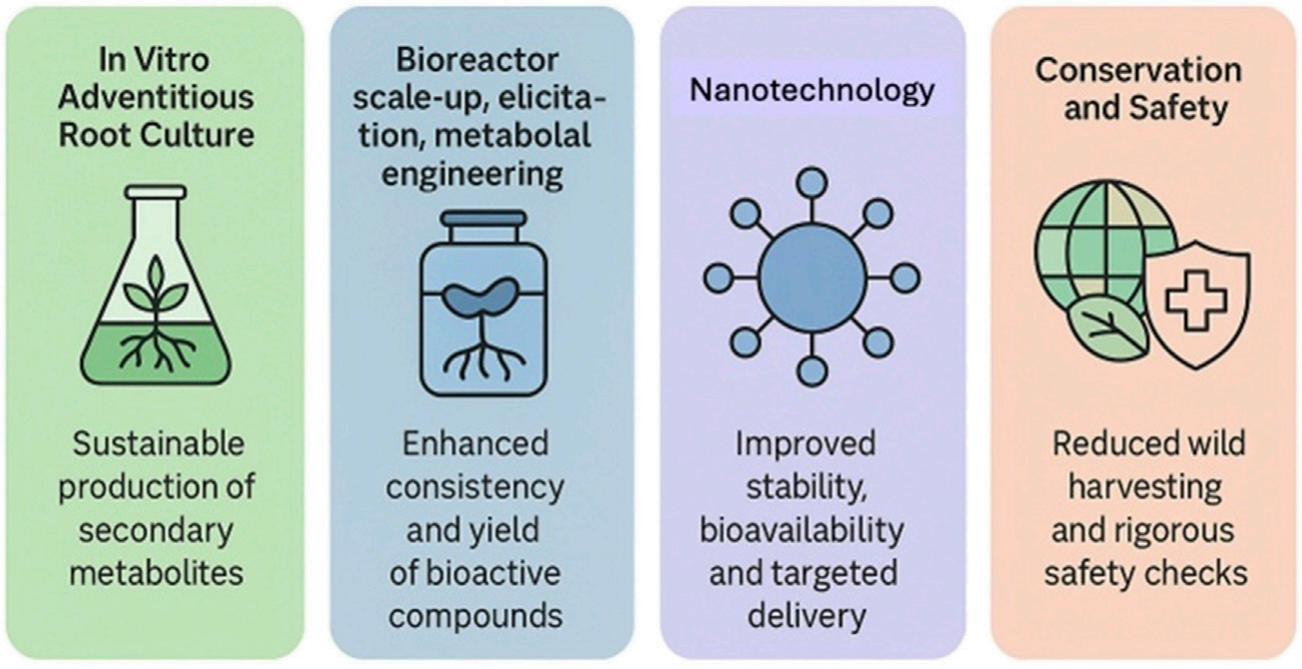
Sequential Integration of Advanced Production Technologies. Sequential schematic showing the continuum from *in vitro* adventitious-root culture, bioreactor scale-up, elicitation, and metabolic-engineering processes toward nanotechnology-based formulation. Each step represents an incremental enhancement in consistency, scalability, and sustainability for herbal-medicine production aligned with Good Agricultural, Manufacturing, and Laboratory Practices. Abbreviations: GACP, Good Agricultural and Collection Practices; GMP, Good Manufacturing Practices; GLP, Good Laboratory Practices; QbD, Quality by Design.

Collectively, these technologies reinforce the triad of consistency, safety, and conservation in herbal medicine production. The combination of *in vitro* cultivation and controlled bioprocessing minimizes variability while reducing environmental pressure on natural populations (Ogidi et al., 2024; Tamil Selvi and Srinivas, 2017). Nanotechnology, meanwhile, strengthens formulation reliability and therapeutic precision. Yet, achieving full regulatory compliance requires systematic safety evaluation, transparent traceability, and validated analytical standards (Govindaraghavan and Sucher, 2015; Osman et al., 2023). The convergence of biotechnology, metabolic design, and nanoscience thus marks a pivotal transition from traditional craftsmanship toward a scientifically standardized herbal industry capable of meeting global regulatory expectations and contributing to sustainable healthcare innovation. These technological advances provide regulatory agencies such as BPOM with a scientific foundation to transition from descriptive assessments of herbal quality toward predictive, process-based quality control. The integration and interrelation of these production technologies are illustrated in Figure 1, which conceptually maps their sequential contribution to quality, sustainability, and regulatory alignment within the evidence-based production framework.

## 4 Omics-based quality control and authentication

Omics-based technologies have transformed the landscape of quality assurance for herbal medicines by bridging molecular identification, chemical profiling, and process design into a single, evidence-driven framework. The use of DNA barcoding has become a widely adopted tool for supporting the authentication of botanical ingredients, employing short and standardized genomic loci such as Ribulose-1,5-bisphosphate carboxylase/oxygenase large subunit (rbcL), Maturase K (matK), and Internal Transcribed Spacer (ITS) to assist in species-level identification where reference databases and laboratory capability permit, while in other contexts providing reliable genus-level resolution (Patel et al., 2024; Shreedevasena et al., 2024). This molecular approach effectively detects adulteration, contamination, and substitution problems that have historically compromised the credibility and therapeutic reliability of herbal preparations (Kreuzer et al., 2019; Nazar et al., 2025). The inclusion of DNA barcoding protocols in pharmacopoeial guidelines and regulatory frameworks has further strengthened its position as a cornerstone of industrial quality control (Sgamma et al., 2017). Complementing this, DNA metabarcoding uses high-throughput sequencing to simultaneously identify multiple species within complex herbal mixtures, offering superior sensitivity in distinguishing intended ingredients from contaminants (Liu et al., 2019; Wu and Shaw, 2022). This method has proven particularly valuable in large-scale surveillance of multi-component products, enabling regulatory authorities to enforce authenticity and traceability standards in both domestic and international markets (De Boer et al., 2015).

Beyond genetic authentication, metabolomics provides a chemical dimension to herbal quality assessment by generating comprehensive metabolic fingerprints that reflect the biochemical identity and therapeutic potential of plant materials. Analytical platforms such as liquid chromatography–mass spectrometry (LC–MS), gas chromatography–mass spectrometry (GC–MS), and nuclear magnetic resonance (NMR) spectroscopy allow for global profiling of metabolites, ensuring batch-to-batch consistency and pharmacological reproducibility (Alum et al., 2025; Lee et al., 2017). These methods not only quantify marker compounds but also uncover subtle variations in phytochemical composition that can influence clinical performance (García-Pérez et al., 2024). The application of chemometrics, including principal component analysis (PCA) and partial least squares–discriminant analysis (PLS-DA), enables data-driven interpretation of complex metabolic datasets, facilitating the classification of species, detection of adulteration, and assessment of geographical origin (Atta et al., 2023; Kusumadewi et al., 2022). This integration of analytical chemistry and statistical modelling transforms metabolomics into a powerful instrument for both authentication and standardization, aligning scientific rigor with regulatory expectations.

The convergence of omics and Quality by Design (QbD) principles marks a decisive advancement in the reproducibility of herbal medicine production. QbD emphasizes proactive quality assurance through the identification of critical quality attributes (CQAs) and the establishment of design spaces that define acceptable variability during manufacturing (Indrayanto, 2018). Incorporating DNA and metabolomic data into this framework provides quantitative parameters for controlling raw-material diversity and process fluctuations, thereby reducing uncertainty in final product performance (Rashid et al., 2025). Multivariate statistical modelling and machine learning algorithms further refine this approach, transforming omics-derived datasets into predictive tools for process optimization and risk mitigation (Ibrahim et al., 2024). As a result, production shifts from retrospective compliance to dynamic control, embedding scientific evidence at every stage of the manufacturing cycle. The complementary roles of each omics dimension within the integrated quality framework are summarized in Table 3, highlighting their analytical methods, regulatory functions, and contribution to reproducibility.

**TABLE 3:** Functional integration of omics approaches for herbal medicine quality control.

Omics dimension	Analytical method	Primary output	Regulatory function	Quality attribute strengthened
DNA Barcoding	PCR amplification of *matK*, *rbcL*, ITS regions	Verified genetic identity of species	Authentication and prevention of substitution or adulteration	Identity and Traceability
DNA Metabarcoding	High-throughput sequencing (Illumina, Ion Torrent)	Detection of multi-species components in complex products	Surveillance of multi-ingredient formulations	Integrity and Transparency
Metabolomics	LC–MS, GC–MS, NMR profiling	Comprehensive metabolite fingerprint and bioactive quantification	Standardization and batch consistency	Chemical Consistency
Chemometrics	PCA, PLS-DA, cluster modeling	Multivariate classification and pattern recognition	Predictive modelling for quality monitoring	Reproducibility and Differentiation
Omics × QbD Integration	Multivariate design space modeling	Predictive process control and tolerance setting	Evidence-based regulatory inspection	Manufacturing Robustness
Two-Key Batch Release (DNA × Metabolite)	Dual verification workflow	Genomic–metabolomic concordance score	Final release certification	Regulatory Assurance and Safety

This table presents the functional roles of DNA, barcoding, metabarcoding, metabolomics, chemometrics, and QbD-modelling in herbal quality control, including analytical methods, regulatory functions, and strengthened quality attributes.

Abbreviations: DNA, deoxyribonucleic acid; LC–MS, Liquid Chromatography–Mass Spectrometry; GC–MS, Gas Chromatography–Mass Spectrometry; NMR, nuclear magnetic resonance; PCA, principal component analysis; PLS-DA, Partial Least Squares–Discriminant Analysis; QbD, quality by design.

**FIGURE 2:**
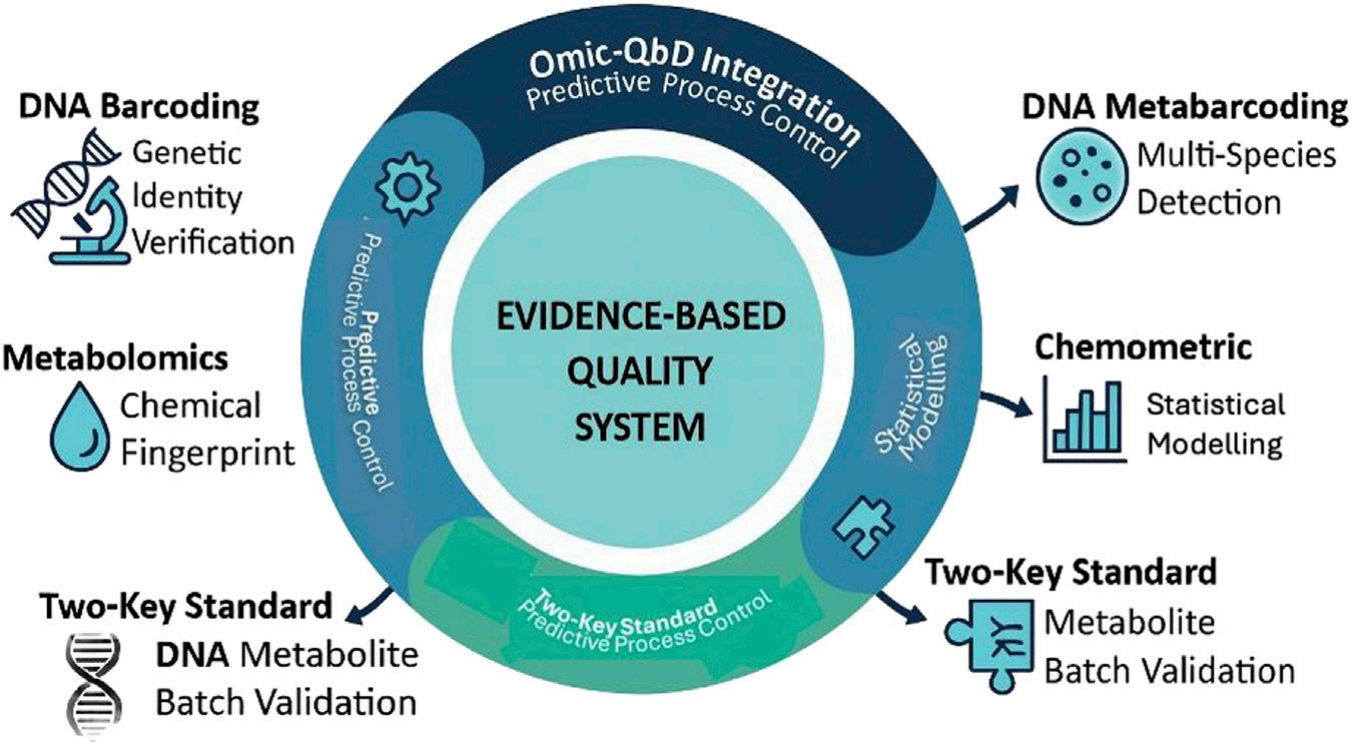
Dual-Omics Authentication and Quality-by-Design Workflow. Overview of the proposed “two-key” batch-release system combining DNA barcoding/metabarcoding and metabolomics with chemometric analysis. The figure illustrates how omics data are validated under GLP conditions to ensure species authenticity, chemical consistency, and traceability from raw material to finished product. Abbreviations: DNA, Deoxyribonucleic Acid; GLP, Good Laboratory Practices; PCA, Principal Component Analysis; PLS-DA, Partial Least Squares–Discriminant Analysis; QbD, Quality by Design.

A forward-looking concept within this paradigm is the two-key batch-release specification, which integrates genomic and metabolomic verification as dual criteria for quality assurance. In this system, the first “key” ensures genetic authenticity via DNA barcoding or metabarcoding, while the second verifies chemical integrity through metabolite profiling and chemometric validation (Raclariu et al., 2018; Kumar et al., 2023). Only batches that meet both identity and compositional thresholds are approved for release, ensuring that products reaching consumers are both authentic and consistent in bioactive content. This dual-omics standard transforms herbal quality evaluation from a fragmented to a holistic framework linking molecular evidence, chemical consistency, and regulatory accountability. It represents a critical evolution toward globally harmonized quality systems that combine technological precision with public health protection. Integrating omics-based authentication into regulatory workflows ensures molecular traceability and aligns Indonesia’s framework with WHO recommendations for herbal pharmacovigilance and batch-level transparency. The interconnection between molecular, chemical, and design-based quality dimensions is illustrated in Figure 2, presenting the integrative omics framework that underpins evidence-based authentication and batch reproducibility in herbal medicines.

## 5 Regulatory harmonization and policy framework

### 5.1 Global reference frameworks

Global regulatory convergence for herbal medicines has evolved unevenly across jurisdictions, reflecting differing policy priorities, institutional capacities, and cultural contexts. The European Union (EU) represents the most advanced model, where harmonized directives integrate the European Pharmacopoeia, herbal monographs, and traditional-use registration pathways to ensure consistent safety and efficacy evaluations (Knoess and Wiesner, 2019; Qu et al., 2018). This system not only streamlines market authorization but also builds public trust by mandating evidence-based validation. In contrast, India’s regulatory framework despite its strong ethnopharmacological heritage still faces structural gaps, including limited enforcement of GMP and fragmented quality standards, which hinder the international competitiveness of Ayurvedic and Siddha products (Dubale et al., 2025; Parveen et al., 2015). The WHO provides overarching technical guidance, emphasizing the formulation of national policies, herbal pharmacopoeias, and standardized quality-assurance protocols to safeguard efficacy and safety in global trade (Kosoe et al., 2024). Collectively, these frameworks illustrate how regional diversity can coexist with a shared ambition for regulatory coherence and consumer protection. The comparative characteristics of major global and regional regulatory systems are summarized in Table 4, outlining their key strengths, limitations, and relevance to Indonesia’s evolving herbal medicine framework.

**TABLE 4:** Comparative framework of global and regional herbal regulations.

Jurisdiction/Organization	Core regulatory reference	Key characteristics	Strengths	Challenges	Relevance to Indonesia (BPOM)
European Union (EU)	*Directive 2004/24/EC*, European Pharmacopoeia, EU Herbal Monographs	Harmonized legislation with unified safety, efficacy, and quality requirements	Streamlined authorization, mutual recognition, transparent monograph system	Limited flexibility for region-specific traditional systems	Serves as benchmark for structured registration and GMP enforcement
India	*Drugs and Cosmetics Act (Ayurveda, Siddha, Unani)*	National pharmacopoeia with decentralized oversight	Strong traditional base and innovation potential	Weak GMP implementation, inconsistent standards	Highlights need for stronger enforcement and traceability
World Health Organization (WHO)	*WHO Guidelines for Quality Assurance of Herbal Medicines*	Global technical reference supporting national policy development	Universal framework promoting safety and efficacy	Requires localization and national adaptation	Foundation for BPOM’s evidence-based regulation and ASEAN harmonization
ASEAN	*ASEAN Harmonized Cosmetic and Herbal Standards*	Regional effort toward shared technical requirements and labelling standards	Facilitates trade and standard alignment	Varying implementation capacities among members	BPOM’s active role as coordinator for herbal harmonization
Indonesia (BPOM)	*PerBPOM Regulations, ASEAN Herbal Harmonization Framework*	Integration of GACP–GMP–GLP; participation in WHO IRCH	Aligns local traditional heritage with international quality systems	Resource disparities and laboratory standardization	Model of “loose harmonization” balancing regulation and biodiversity preservation

This table compares global and regional regulatory frameworks including those of the EU, india, WHO, ASEAN, and Indonesia highlighting their key characteristics, strengths, and relevance to Indonesia’s regulatory development.

Abbreviations: WHO, world health organization; IRCH, international regulatory cooperation for herbal medicines; BPOM, indonesian food and drug authority; ASEAN, association of southeast asian nations.

Building upon these global foundations, the next layer of regulatory alignment emerges at the regional level, where countries coordinate standards to facilitate mutual recognition and cross-border trade.

### 5.2 Regional alignment through ASEAN harmonization

Within this global matrix, the integration of GACP, GMP, and GLP has emerged as a foundational triad to ensure the traceability, reproducibility, and safety of herbal medicinal products. The European model enforces this continuum from cultivation to laboratory verification, providing a replicable template for national regulators (Dubale et al., 2025). In the Indonesian context, the Indonesian Food and Drug Authority (BPOM) has progressively adopted this integrated approach, linking agricultural sourcing, processing, and laboratory analytics under unified regulatory oversight. Such cross-practice alignment is pivotal for guaranteeing product uniformity and enabling compliance with both domestic and ASEAN market-entry requirements. This holistic chain of quality assurance from GACP through GMP to GLP creates the infrastructural backbone for evidence-based production systems and facilitates mutual recognition across borders.

As regional frameworks strengthen technical coherence, national regulatory systems must articulate their internal classification structures to ensure alignment with international expectations.

### 5.3 National regulatory categories of herbal medicines in Indonesia

Indonesia classifies herbal medicinal products into three regulatory pathways based on the level of evidence required for safety, quality, and efficacy.

1. Jamu (Traditional Herbal Medicines) – Products based on long-standing empirical use. These require compliance with safety, quality, and labelling standards but do not require preclinical or clinical evidence.
2. Obat Herbal Terstandar (OHT–Standardized Herbal Medicines) – Products formulated from standardized extracts and supported by preclinical studies demonstrating safety and efficacy. Compliance with Good Agricultural and Collection Practices and Good Manufacturing Practices is required.
3. Fitofarmaka (Phytopharmaceuticals) – The highest regulatory category, requiring comprehensive clinical evidence, validated standardized extracts, and full non-clinical and clinical documentation. Fitofarmaka are evaluated similarly to modern medicines in terms of regulatory rigor.

The main regulatory instruments governing these three product categories are summarized in Table 5.

**TABLE 5:** Indonesian regulatory framework for herbal medicines.

Regulation/Document	Purpose/Scope	Key requirements	Product category
PerBPOM No. 29/2023 – Traditional Medicine Quality Standards	Sets quality, safety, and labeling requirements	Microbial limits, heavy metals, stability, labeling	Jamu, OHT, Fitofarmaka
PerBPOM No. 8/2024 – Clinical Trial	Defines evidence and evaluation requirements	Non-clinical and clinical data, validated extracts, GMP	Fitofarmaka
PerBPOM No. 25/2021 – CPOTB	Good Manufacturing Practices for herbal medicines	Facility compliance, process validation, QA/QC	Jamu, OHT, Fitofarmaka
Farmakope Herbal Indonesia (FHI)	Provides monographs and analytical standards	Identity, purity, assay methods	OHT, Fitofarmaka
Formularium Obat Herbal Indonesia (FOHI)	Clinical listing and therapeutic indications	Evidence-based clinical inclusion	Fitofarmaka
ASEAN Traditional Medicines Harmonization Framework	Regional alignment of safety, quality, and documentation	ASEAN technical requirements, labeling standards	All categories

This table summarizes the main national and regional regulatory instruments governing herbal medicines in Indonesia, including regulation numbers, scopes, and key requirements, providing a structured overview of how Jamu, OHT, and Fitofarmaka are regulated within the BPOM, and ASEAN, harmonization context.

Abbreviations: BPOM, indonesian food and drug authority; OHT, obat herbal terstandar; GMP, good manufacturing practices; CPOTB, cara pembuatan obat tradisional yang baik; FHI, farmakope herbal indonesia; FOHI, formularium obat herbal indonesia; ASEAN, association of southeast asian nations.

These nationally defined categories position Indonesia to engage more strategically in regional and global harmonization efforts, particularly as regulatory cooperation intensifies across ASEAN, and WHO, platforms.

### 5.4 Strategic pathways for regulatory convergence

Indonesia’s proactive participation in the ASEAN Herbal Harmonization initiative and the WHO International Regulatory Cooperation for Herbal Medicines (IRCH) underscores its strategic role in shaping regional and global standards (Rahman et al., 2025). As one of the most biodiverse nations, Indonesia contributes not only raw materials but also technical expertise in monograph development, analytical validation, and risk-based regulatory approaches. These contributions strengthen ASEAN’s collective framework for herbal product assessment, ensuring that safety, quality, and efficacy principles remain aligned with WHO’s global benchmarks. Moreover, Indonesia’s engagement demonstrates how regional collaboration can serve as an instrument of soft diplomacy bridging scientific evidence, economic policy, and traditional knowledge to enhance equitable access to safe herbal therapies.

**FIGURE 3:**
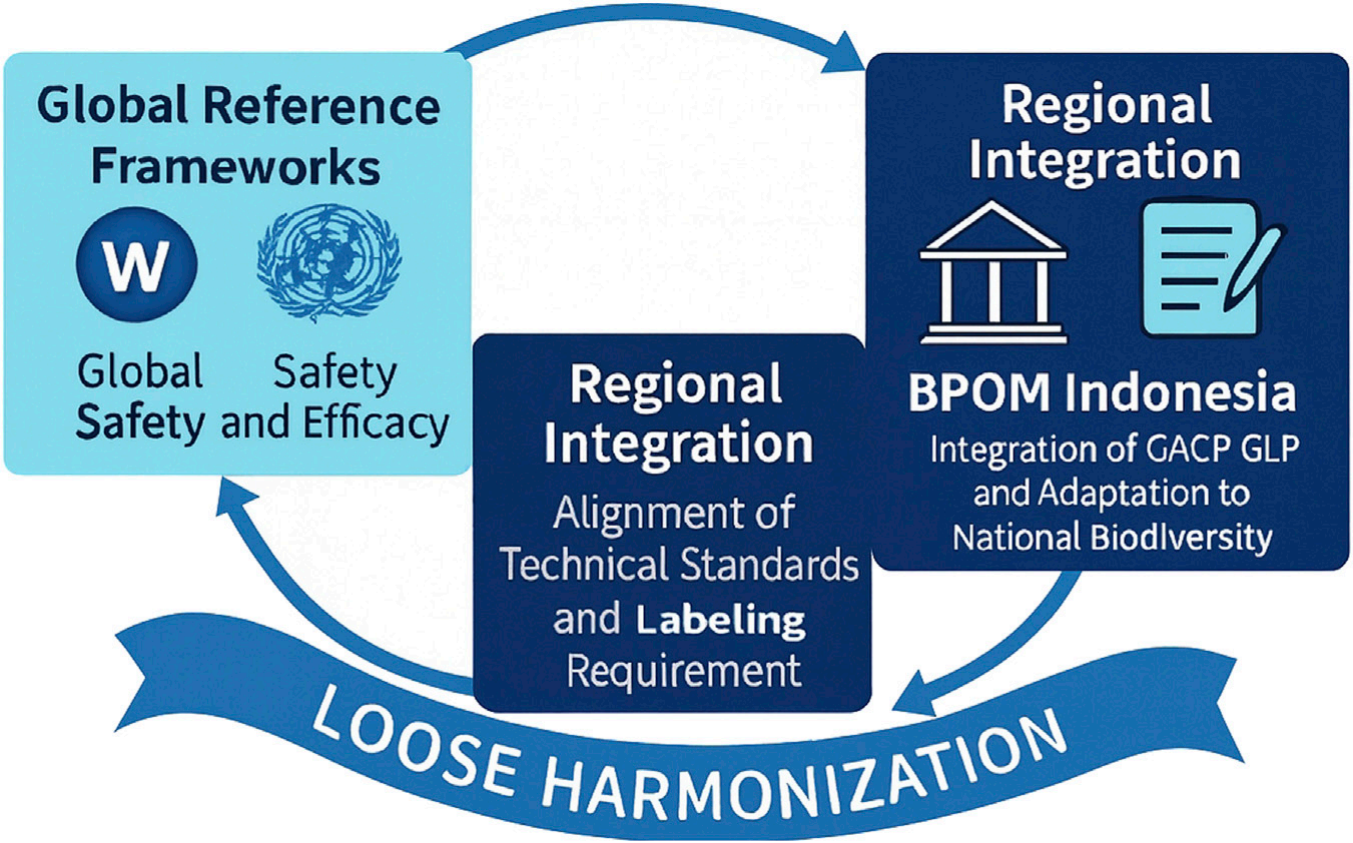
Multi-Level Regulatory Harmonization Framework. Conceptual map of global–regional–national alignment: WHO (IRCH/GBT/WLA) → ASEAN harmonization initiatives → Indonesia’s BPOM regulatory system. The arrows demonstrate information flow, reliance mechanisms, and capacity-building loops enabling “loose harmonization” that maintains cultural and biodiversity diversity while achieving regulatory convergence. Abbreviations: WHO, World Health Organization; IRCH, International Regulatory Cooperation for Herbal Medicines; BPOM, Indonesian Food and Drug Authority.

The evolving paradigm of “loose harmonization” offers a pragmatic solution to balancing standardization with cultural and biological diversity. Unlike rigid alignment, this approach allows regulatory equivalence through shared principles rather than uniform procedures, accommodating local variations in traditional medical systems such as Ayurveda, Jamu, and Traditional Chinese Medicine (Knoess and Wiesner, 2019; Sharma, 2025). It fosters regulatory flexibility encouraging innovation, biodiversity utilization, and trade while maintaining core safety and quality parameters consistent with WHO guidance (Kosoe et al., 2024). For Indonesia, this model aligns well with BPOM’s policy direction: pursuing regulatory alignment within ASEAN while safeguarding indigenous pharmacopoeia identity and local manufacturing practices. Thus, *loose harmonization* emerges not as a compromise but as a strategic form of regulatory pluralism where practical alignment coexists with the preservation of national heritage, scientific integrity, and market inclusivity. This harmonization trajectory empowers BPOM to operationalize international best practices while safeguarding national biodiversity, exemplifying a balanced model of regulatory convergence and cultural integrity. The multilevel interactions among global, regional, and national regulatory systems are illustrated in Figure 3, highlighting Indonesia’s role in achieving practical alignment through the principle of loose harmonization.

## 6 Integration into healthcare systems and pharmacovigilance

The clinical integration of herbal medicines within modern healthcare increasingly depends on the principles of evidence-based medicine (EBM), which synthesizes clinical expertise, patient preferences, and rigorously validated research to inform therapeutic decisions. Structured care pathways and clinical guidelines have demonstrated effectiveness in standardizing practice and improving treatment outcomes by embedding phytotherapeutics into mainstream clinical workflows (Roberts et al., 2022). Nevertheless, implementation remains uneven due to gaps in robust clinical data, heterogeneous study designs, and limited translational evidence connecting traditional formulations with biomedical endpoints (Melzer, 2017). These challenges are compounded by differing epistemological frameworks between traditional and allopathic systems, which complicate regulatory recognition and reimbursement mechanisms. To ensure equitable and safe integration, future pathways must emphasize high-quality clinical trials, standardized dosing, and the development of hybrid models that respect cultural context while adhering to modern scientific rigor. Such alignment not only advances therapeutic credibility but also strengthens the legitimacy of herbal medicine as a validated component of evidence-based healthcare. The multidimensional components linking clinical evidence, pharmacovigilance, digital systems, and policy frameworks are summarized in Table 6, outlining their mechanisms, outcomes, and regulatory implications for healthcare integration.

**TABLE 6:** Evidence–surveillance–policy framework for integrating herbal medicines into healthcare systems.

Domain	Key focus	Mechanism/Tools	Outcome/Impact	References
Clinical Integration	Evidence-based use of herbal medicines through guidelines and care pathways	Clinical trials, meta-analyses, electronic medical records (EMR) pathways	Reduced care variability, enhanced therapeutic outcomes	Roberts et al. (2022)
Pharmacovigilance Models	Detection and monitoring of herbal adverse reactions	Patient reporting, HDI surveillance, pharmacogenetic screening	Early signal detection, improved safety response	Barnes et al. (2016), Pai et al. (2024), Shaw et al. (2012)
Real-World Evidence (RWE)	Continuous post-market validation of safety and efficacy	National registries, AI-based signal analytics, data interoperability	Informed policy decisions, predictive risk modelling	Hölzle et al. (2024), Habs et al. (2023), Hennessy et al. (2025)
Digital Platforms	Infrastructure for integrated data collection and monitoring	E-reporting portals, mHealth apps, automated databases	Real-time data flow, stakeholder transparency	Petcharat and Nachin (2024), Wittayakhom et al. (2024)
Policy and Governance	Harmonized standards ensuring safety, affordability, and trust	WHO guidance, public education, labelling regulation	Strengthened global pharmacovigilance and public confidence	Sethi et al. (2025), Zarsuelo et al. (2018)

This table summarizes the components linking clinical integration, pharmacovigilance, real-world evidence, digital systems, and policy mechanisms in supporting safe and evidence-informed use of herbal medicines.

Abbreviations: EMR, electronic medical records; HDI, Herb–Drug Interaction; AI, artificial intelligence.

Conventional pharmacovigilance frameworks originally structured for single-compound synthetic drugs struggle to capture the complexity of herbal medicines, which often comprise multiple bioactive constituents and variable compositions. This heterogeneity complicates signal detection and causality assessment, leading to persistent underreporting of adverse drug reactions (ADRs) and inconsistent documentation across regions (Shaw et al., 2012). Moreover, the lack of standardized reporting tools and insufficient clinician awareness further weakens surveillance capacity (Sethi et al., 2025). To address these deficiencies, contemporary models advocate for patient-centered reporting mechanisms, integration of pharmacogenetic insights to predict idiosyncratic responses, and the development of specialized data analytics tailored for herbal ADR detection (Pai et al., 2024; Ranjan et al., 2021). Strengthening regulatory infrastructure through digital monitoring platforms and stakeholder training is equally essential to enhance data quality and responsiveness. Collectively, these innovations signify a transition from passive, post-market reporting toward an adaptive, risk-based pharmacovigilance ecosystem capable of ensuring both the safety and credibility of evidence-based herbal therapies.

Real-world evidence (RWE) has emerged as a critical complement to randomized controlled trials in assessing the effectiveness and safety of herbal medicines within diverse patient populations. By capturing data from actual clinical settings, RWE bridges the gap between experimental efficacy and routine therapeutic performance, allowing for a more comprehensive understanding of long-term outcomes and herb–drug interactions (Hölzle et al., 2024; Habs et al., 2023; Hennessy et al., 2025; Symma et al., 2025). The growing integration of digital registries, mobile health applications, and AI-driven reporting systems now enables real-time pharmacovigilance and outcome monitoring, enhancing data precision and temporal responsiveness (Petcharat and Nachin, 2024; Wittayakhom et al., 2024). Such digital infrastructures not only strengthen post-market surveillance but also support early signal detection and predictive risk modeling by consolidating large-scale, multidimensional datasets (Suriyapaiboonwattana et al., 2025). Collectively, these innovations transform pharmacovigilance from a reactive system into an adaptive, data-driven ecosystem one capable of continuously improving herbal medicine safety, regulatory oversight, and patient trust through transparent, evidence-based insight generation.

**FIGURE 4:**
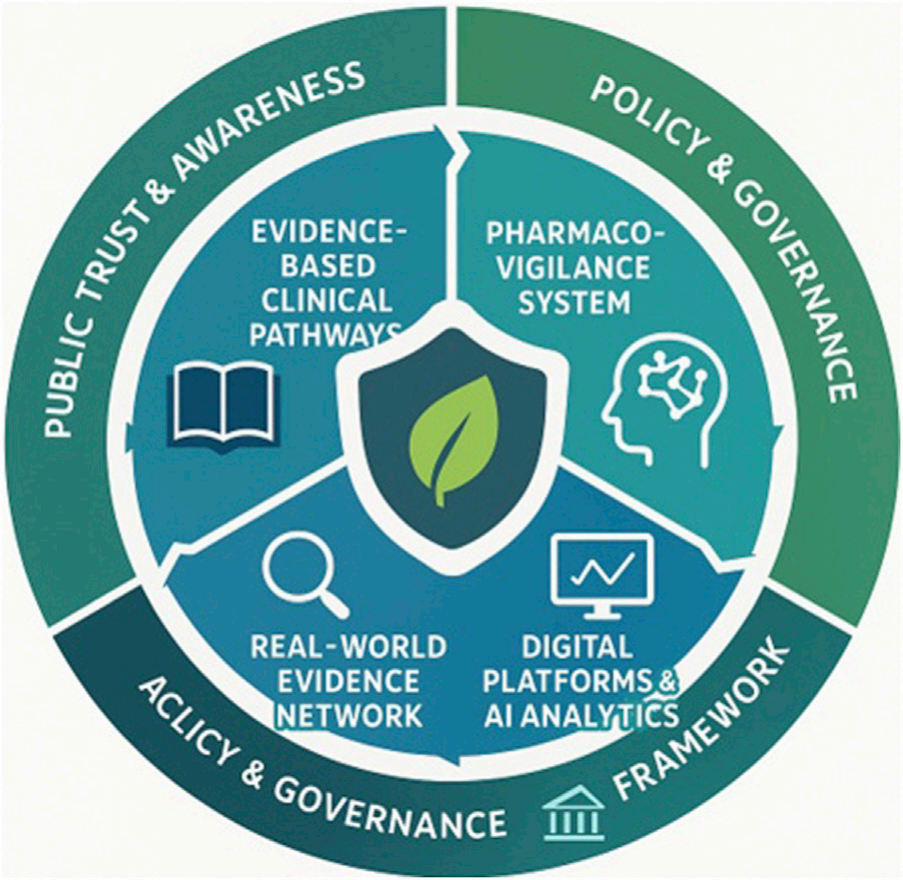
Integrated Framework for Herbal-Medicine Safety and Healthcare Adoption. Comprehensive framework connecting production, quality control, regulation, pharmacovigilance, and healthcare integration. The model shows how validated omics workflows, digital traceability, and real-world-evidence monitoring strengthen BPOM’s evidence-based governance and public-health resilience. Abbreviations: PV, Pharmacovigilance; RWE, Real-World Evidence; AI, Artificial Intelligence.

Robust regulatory frameworks remain fundamental to ensuring the safety, efficacy, and equitable accessibility of herbal medicines. Effective governance requires the implementation of stringent quality control, transparent labelling, and continuous post-market surveillance to prevent adulteration and misinformation (Zarsuelo et al., 2018; Mssusa et al., 2023). However, regulation alone is insufficient without parallel investment in education and stakeholder engagement. Enhancing literacy among healthcare professionals and the public about the proper use, benefits, and potential risks of herbal products is critical to improving adverse event reporting and fostering a culture of shared accountability in pharmacovigilance (Hasen and Hashim, 2021; Mssusa et al., 2025). Globally, harmonization efforts guided by the World Health Organization are promoting convergence of safety monitoring systems, evidence standards, and labelling practices, enabling data interoperability and mutual recognition across jurisdictions (Hennessy et al., 2025; Sethi et al., 2025). Such coordinated regulation and transparent communication strengthen not only pharmacovigilance capacity but also public trust ensuring that the growing demand for herbal medicines is matched by credible oversight, affordability, and ethical stewardship. Embedding these pharmacovigilance and real-world evidence mechanisms into Indonesia’s healthcare regulation will enhance patient safety, affordability, and public confidence in herbal medicine oversight. The interactive relationship among clinical practice, pharmacovigilance, real-world data, and policy harmonization is illustrated in Figure 4, demonstrating how evidence and safety data flow within an integrated healthcare framework.

## 7 Policy and research implications

BPOM’s evolving mandate reflects an urgent need to build a regulatory–scientific ecosystem that balances public protection with innovation and competitiveness across Indonesia’s pharmaceutical and food industries. Strengthening this integration requires a shift from traditional compliance enforcement toward a more adaptive, collaborative, and evidence-based governance model. As discussed by Kashuri and Ikrar (2025), BPOM’s strategic approach must connect regulatory supervision with the empowerment of small and medium enterprises through multi-stakeholder collaboration among academia, industry, and government agencies (Kashuri and Ikrar, 2025). By adopting flexible, technology-enabled, and results-oriented regulatory mechanisms, the agency can foster innovation while maintaining rigorous safety and quality standards. This model aligns with global regulatory science trends that emphasize transparency, data-driven risk assessment, and policy agility. Embedding scientific evidence within regulatory processes not only enhances decision-making efficiency but also improves public trust and industry accountability. In doing so, BPOM can evolve into a knowledge-driven regulator that bridges science and policy promoting both consumer safety and sustainable innovation in Indonesia’s growing health and bioeconomy sectors.

Establishing validated omics workflows within regulatory laboratories is essential to ensure analytical transparency, reproducibility, and scientific credibility in data-driven decision-making. Previous inconsistencies in omics analyses particularly those leading to premature clinical trial interpretations have underscored the importance of methodological rigor and traceable data provenance (Zheng et al., 2015). Implementing semantic workflow systems, such as the WINGS platform, provides a structured solution that enhances reproducibility by linking analytical steps, metadata, and computational processes in a verifiable manner. This digital traceability not only strengthens scientific reliability but also supports regulatory accountability by allowing independent verification of analytical validity. To sustain such standards, the adoption of GLP-compliant frameworks for omics technologies has become both feasible and imperative. GLP-based quality assurance promotes standardized data acquisition, controlled instrument calibration, and method validation, thereby bridging the gap between research-grade and regulatory-grade data (Kauffmann et al., 2017; Marx-Stoelting et al., 2015; Sauer et al., 2017). Collectively, these measures establish a foundation for integrating omics into regulatory toxicology and pharmacovigilance, enabling BPOM and other authorities to adopt molecular evidence as a credible pillar of evidence-based regulation.

Collaboration with international and regional bodies such as the WHO and ASEAN is pivotal for aligning regulatory standards, strengthening institutional capacity, and promoting mutual recognition of regulatory decisions. The WHO’s Global Benchmarking Tool (GBT) and WHO-Listed Authority (WLA) frameworks have provided structured mechanisms for assessing national regulatory maturity, facilitating reliance pathways, and accelerating access to quality-assured medical and herbal products through transparent, evidence-based evaluation systems (Broojerdi et al., 2024). For Indonesia’s BPOM, engagement with WHO’s International Regulatory Cooperation for Herbal Medicines (IRCH) serves as a strategic platform for exchanging scientific data, benchmarking performance, and harmonizing inspection and post-market surveillance practices. At the regional level, ASEAN’s initiatives to harmonize labor, industrial, and food safety standards demonstrate the value of shared regulatory frameworks in reducing technical barriers and promoting equitable market access across member states (Li et al., 2022; Sale, 2020). This dual collaboration global through WHO and regional through ASEAN creates a foundation for regulatory convergence that balances international best practices with national priorities. It enables BPOM and its counterparts to build trust, optimize resources, and advance collective resilience in ensuring the safety, efficacy, and quality of traditional and modern health products.

Ensuring fair and equitable benefit-sharing and intellectual property (IP) protection is vital to balancing innovation, community rights, and biodiversity sustainability. Despite growing international recognition, the implementation of benefit-sharing frameworks continues to face challenges, particularly in translating global legal instruments into actionable national policies that effectively safeguard traditional knowledge and indigenous innovation (Morgera, 2024; Willcox et al., 2015). Many benefit-sharing agreements remain fragmented, with limited mechanisms for recognizing local custodians or ensuring that profits from bioprospecting are reinvested into conservation and community welfare (Morrison et al., 2021). A holistic approach that integrates legal reform, ethical IP governance, and participatory resource management is therefore essential. Recent studies emphasize that sustainable benefit-sharing can only be achieved through inclusive engagement with local stakeholders and transparent governance structures that value traditional ecological knowledge as part of national innovation systems (Penteado et al., 2024; Vijayasree et al., 2024). For BPOM, embedding these principles into regulatory and research frameworks would not only promote equitable access and sustainability but also strengthen Indonesia’s leadership in aligning biodiversity conservation with responsible innovation.

Building robust cross-sector partnerships among academia, industry, and regulatory authorities is fundamental to accelerating the translation of scientific discoveries into effective regulatory and policy frameworks. Collaborative models, such as the CLARITY-BPA program, demonstrate how coordinated research involving academic institutions, government agencies, and private sector experts can enhance data transparency, methodological consistency, and mutual trust in evidence interpretation (Kashuri and Ikrar, 2025). These collaborations enable regulators to access cutting-edge methodologies while allowing scientists to align research outputs with policy relevance and risk-assessment needs. For BPOM, fostering similar collaborative ecosystems can help bridge gaps between laboratory innovation and regulatory application, particularly in areas such as safety evaluation, omics data validation, and herbal product standardization. Such partnerships also promote open science, reduce duplication of effort, and strengthen decision-making through shared databases and co-designed research protocols. Ultimately, a sustained triad of academia–industry–regulator cooperation can transform regulatory science into a dynamic, evidence-informed discipline enhancing public health protection, regulatory agility, and national competitiveness in biomedical and natural product innovation.

Embedding EBP within national regulatory frameworks strengthens the scientific basis of decision-making, aligns local practices with WHO and ASEAN harmonization agendas, and enhances public trust in traditional medicines. By linking regulatory science with health system resilience, Indonesia can serve as a regional hub for regulatory capacity building and evidence-based policy implementation.

## 8 Future directions in herbal medicine

The establishment of a national reference database that integrates DNA sequencing with metabolomics profiling represents a transformative foundation for scientific regulation and personalized herbal medicine. By linking genomic, proteomic, and metabolomic information, this database would enable precise authentication of raw herbal materials, identification of biomarkers, and enhanced understanding of metabolic pathways related to therapeutic efficacy. Drawing on global initiatives such as the *Human Metabolome Project (HMP)*, Indonesia could develop a harmonized repository that connects genetic determinants with chemical fingerprints to ensure the consistency and authenticity of herbal ingredients (Aristizabal-Henao et al., 2021; Vijay et al., 2024). Such a database would not only support research and innovation but also serve as a regulatory instrument for BPOM, allowing real-time verification of herbal product integrity, strengthening traceability systems, and aligning national standards with international scientific frameworks.

Developing real-world evidence (RWE) registries is essential for building a comprehensive herbal pharmacovigilance system that monitors the safety, quality, and therapeutic performance of herbal medicines in real-life settings. Unlike controlled clinical trials, RWE registries capture data from diverse populations, enabling regulators to detect adverse events, herb–drug interactions, and patterns of use across demographic and geographic variations (Habs et al., 2023; Ekhart et al., 2025). Incorporating molecular tools such as DNA barcoding would enhance the reliability of these databases by confirming the botanical identity of herbal products (De Boer et al., 2015). For BPOM, integrating RWE systems with post-market surveillance platforms would facilitate evidence-based decision-making, improve safety monitoring, and strengthen public confidence in the nation’s herbal medicine ecosystem, aligning with WHO’s global pharmacovigilance vision.

Promoting international validation studies for QbD frameworks in herbal medicine manufacturing is crucial to achieving global harmonization of quality standards. QbD provides a scientific foundation for defining and controlling critical quality attributes (CQAs) to ensure product safety, efficacy, and reproducibility (Devangan et al., 2024; Mohammed et al., 2015). However, challenges persist due to the intrinsic variability of botanical raw materials and diverse regional regulatory requirements. Collaborative validation studies across ASEAN and other regulatory regions can establish shared benchmarks for process control, analytical verification, and batch-to-batch consistency (Roy and Gupta, 2021). For Indonesia’s BPOM, leading such efforts would position the country as a regional hub for regulatory science and reinforce its contribution to ASEAN harmonization, strengthening its role in global herbal standardization.

The 2025–2030 roadmap should focus on institutionalizing evidence-based practice (EBP) through a coordinated framework that integrates regulation, professional training, and intersectoral collaboration. EBP ensures that clinical and policy decisions in herbal medicine are grounded in the best available scientific evidence, improving patient outcomes and healthcare efficiency (Craig et al., 2025; Duff et al., 2025). Structured programs such as *HELIX* ^*4*^ and the *Clinical Scholar Model* have demonstrated effectiveness in strengthening practitioner competence and research literacy (English, 2016). BPOM can adopt similar models to embed EBP into its regulatory ecosystem, ensuring that policies, product approvals, and safety evaluations are consistently supported by empirical data. Institutionalizing EBP through regulation, mentorship, and accreditation will sustain the credibility of Indonesia’s herbal medicine oversight and solidify its leadership in evidence-based regulatory innovation across ASEAN.

## 9 Conclusion

This review underscores that integrating evidence-based production (EBP) into Indonesia’s regulatory system provides a robust scientific foundation for ensuring the quality, safety, and efficacy of herbal medicines. Advances in omics technologies, bioreactor engineering, and nanotechnology collectively enable reproducible, traceable, and sustainable production aligned with Good Agricultural, Manufacturing, and Laboratory Practices. Harmonized standards developed through ASEAN and WHO collaboration position Indonesia to balance regulatory rigor with cultural diversity and biodiversity preservation. Embedding real-world evidence and pharmacovigilance frameworks further strengthens post-market safety monitoring and policy transparency. For BPOM, institutionalizing EBP through cross-sector partnerships, validated analytical workflows, and international cooperation transforms regulation from reactive compliance to proactive governance grounded in data integrity and scientific accountability. Looking forward, Indonesia’s leadership in integrating biotechnology, analytics, and regulatory science will reinforce its global role in shaping the future of herbal medicine regulation and contribute to advancing a resilient, evidence-driven health ecosystem that bridges traditional heritage with modern innovation. Institutionalizing EBP through regulation, digital pharmacovigilance, and international cooperation not only strengthens national oversight but also contributes to global efforts toward equitable, science-driven traditional medicine governance.
